# Supplementary material for: MetaRibo-Seq measures translation in microbiomes
Source: Nat Commun. 2020 Jun 29;11:3268. doi: 10.1038/s41467-020-17081-z (PMC7324362; doi:10.1038/s41467-020-17081-z)
Supplement: Supplementary file 10 — Supplementary Data 7 [file 41467_2020_17081_MOESM10_ESM.zip › File2/Confidence_High_Taxonomy/63206_out.krona.html]

Javascript must be enabled to view this page.

members
magnitude
magnitudeUnassigned
count
unassigned
taxon
rank

63206\_out

1
11

SRS017821\_contig\_number\_23093

2
superkingdom
10

1239
phylum
10

10
186801
class

186802
10
order

SRS104400\_contig\_number\_35908
1

216572
family
6

genus
459786
6


SRS058070\_contig\_number\_92SRS104084\_contig\_number\_9915SRS144506\_contig\_number\_34821SRS148424\_contig\_number\_contig-100\_673.169264
1897011
species
4

2
species
1945593

SRS017191\_contig\_number\_14904SRS021948\_contig\_number\_21138


SRS077294\_contig\_number\_19250SRS149181\_contig\_number\_12659
2
1897045
species

1
family
186803

1
830
genus

1280665
species
1

SRS893300\_contig\_number\_3300
